# Supplementary material for: Post cesarean section surgical site infection and associated factors among women who delivered in public hospitals in Harar city, Eastern Ethiopia: A hospital-based analytic cross-sectional study
Source: PLoS One. 2021 Jun 23;16(6):e0253194. doi: 10.1371/journal.pone.0253194 (PMC8221476; doi:10.1371/journal.pone.0253194)
Supplement: S1 Table — (DOCX) [file pone.0253194.s001.docx]

| Stratification variable | Surgical site infection | | Chi(df) | p-value |
| --- | --- | --- | --- | --- |
|  | Yes | No |  |  |
| Had no co-morbid condition | | | | |
| Less than 7 days hospital stay | 33 | 464 | 25.09 (1) | <0.001 |
| More than 7 days hospital stay | 74 | 357 |  |  |
| Had comorbid conditions | | | | |
| Less than 7 days hospital stay | 8 | 53 | 1.16 (1) | 0.281 |
| More than 7 days hospital stay | 16 | 64 |  |  |

S1 Table. Surgical site infection by hospital stay after cesarean section operation and presence of comorbid conditions
