# Supplementary material for: Post cesarean section surgical site infection and associated factors among women who delivered in public hospitals in Harar city, Eastern Ethiopia: A hospital-based analytic cross-sectional study
Source: PLoS One. 2021 Jun 23;16(6):e0253194. doi: 10.1371/journal.pone.0253194 (PMC8221476; doi:10.1371/journal.pone.0253194)
Supplement: Attachment — Submitted filename: Response to the Editors comments.docx [file pone.0253194.s004.docx]

**Response to comments from the Editor**

Additional Editor Comments (if provided):

I would like to thank the authors for their great effort in significantly improved the quality of their manuscript by answering the comments made by the editor and reviewers. However, the authors need to make the following minor revisions before our acceptance of the manuscript.

- We highly appreciate that the editor valued the changes we have made in the revised manuscript following the comments provided in the first revision. We thank the editor and reviewers for their time and valuable comments and suggestions that improved our manuscript.

1. In Abstract under Results line 4-5 '.......had at least one co-morbid conditions.' should be changed to '.......had at least one co-morbid condition.'

- Thank you for the edits and the suggestion. We have now corrected it in the revised version as: “Emergency CS was conducted for 75.9% (95% CI: 73.2, 78.3) of the women and 13.2% (95% CI: 11.3, 15.4) **had at least one co-morbid condition**.” The changes can be found in lines 32-33 of the revised manuscript.

2. In abstract under Conclusion line 3 '......to avoid unnecessary delays in hospital......' does not make much sense. It should be rewritten as '.......to avoid unnecessary prolonged hospitalization.....'

- Thank you for the edits and the suggestion. We have now corrected it in the revised version as: **“**Screening for preoperative anemia and appropriate correction before surgery**, s**election of the type of anesthesia, **close follow-up to avoid unnecessary prolonged hospitalization**, and careful assessment of membrane status should be considered to avoid preventable SSI and maternal morbidity**.”** The changes can be found in lines 39-42 of the revised manuscript.

3.Under Study area and Period line 29 'from October 11, 2018 and December 31st, 2019' should be changed to 'from October 11, 2018 to December 31, 2019'.

- Thank you for the edits and the suggestion. We have now corrected it in the revised version as: “A fifteen months data, **from October 11, 2018 to December 31, 2019**, …” The changes can be found in line 72 of the revised manuscript.

4. Under Study area and Period line 30 '....during March 1-15/2020 from two public.....'should be changed to '......during March 1 to March 15, 2020 at two public.....'.

- Thank you for the edits and the suggestion. We have now corrected it in the revised version as: “….was extracted from patient records **during March 1 to March 15, 2020 from two public hospitals in Harar city**.” The changes can be found in line 73 of the revised manuscript.

5. Under Data collection line 87 '.......if there is at least one 'yes' response....' should be changed to '.......if there was at least one 'yes' response....'

- Thank you for the edits and the suggestion. We have now corrected it in the revised version as: “A post-CS SSI was defined if there **was** at least one ‘yes’ response to any of the five items within 30 days after operation.” The changes can be found in lines 129-130 of the revised manuscript.

6. Under Demographic and Obstetric Characteristics line 114 '......with most, 78.4%, being under the age of .....' should be rewritten to '......with 65.6% under the age of .....'. In table 1, the number is 65,6%, am i missing something? Please explain.

- Thank you for the edits and the suggestion. We have now corrected it in the revised version as: “The mean age (SD, standard deviation) was 27.4 (±5.3) years **with 65.5% under the age of 30 years and 63.0% were rural residents**.” The changes can be found in lines 156-158 of the revised manuscript.

7. Under Strengths and limitations of the study line232 does BMI mean body mass index? if yes, they should be spelt out.

- Thank you for the edits and the suggestion. We have now corrected it in the revised version as: “important confounding variables (vaginal cleansing before CS, **body mass index**, indications for CS…) might have been missed.” The changes can be found in lines 275 of the revised manuscript.
